# Impact of Autophagy and Aging on Iron Load and Ferritin in *Drosophila* Brain

**DOI:** 10.3389/fcell.2019.00142

**Published:** 2019-07-25

**Authors:** Anne-Claire Jacomin, Kalotina Geraki, Jake Brooks, Vindy Tjendana-Tjhin, Joanna F. Collingwood, Ioannis P. Nezis

**Affiliations:** ^1^School of Life Sciences, University of Warwick, Coventry, United Kingdom; ^2^Diamond Light Source, Harwell Science and Innovation Campus, Didcot, United Kingdom; ^3^School of Engineering, University of Warwick, Coventry, United Kingdom

**Keywords:** aging, autophagy, brain, *Drosophila*, ferritin, iron, synchrotron X-ray fluorescence microscopy

## Abstract

Biometals such as iron, copper, potassium, and zinc are essential regulatory elements of several biological processes. The homeostasis of biometals is often affected in age-related pathologies. Notably, impaired iron metabolism has been linked to several neurodegenerative disorders. Autophagy, an intracellular degradative process dependent on the lysosomes, is involved in the regulation of ferritin and iron levels. Impaired autophagy has been associated with normal pathological aging, and neurodegeneration. Non-mammalian model organisms such as *Drosophila* have proven to be appropriate for the investigation of age-related pathologies. Here, we show that ferritin is expressed in adult *Drosophila* brain and that iron and holoferritin accumulate with aging. At whole-brain level we found no direct relationship between the accumulation of holoferritin and a deficit in autophagy in aged *Drosophila* brain. However, synchrotron X-ray spectromicroscopy revealed an additional spectral feature in the iron-richest region of autophagy-deficient fly brains, consistent with iron–sulfur. This potentially arises from iron–sulfur clusters associated with altered mitochondrial iron homeostasis.

## Introduction

Iron is an essential biometal, widely used as a cofactor by a variety of proteins. Imbalance in iron metabolism, where either a deficiency or excess of iron may have harmful effects, and impaired iron metabolism may be modulators of neurodegeneration in several genetic or sporadic neurodegenerative disorders, such as Alzheimer’s disease, Parkinson’s disease, Huntington’s disease, amyotrophic lateral sclerosis, and multiple sclerosis (Ward et al., 2014; Angelova and Brown, 2015; Biasiotto et al., 2016).

Ferritin is a universal iron storage protein. Two types of subunits, the heavy (H) and light (L) chains, assemble in different ratios into 24-subunit heteropolymers, in which iron can be stored in a mineralized form. Expression of both H and L ferritin chains are closely related to iron bio-availability (Gray and Hentze, 1994). Like in mammals, *Drosophila* genome encodes two types of subunits, known as heavy-chain homolog (Fer1HCH) and light-chain homolog (Fer2LCH) (Georgieva et al., 2002; Nichol et al., 2002; Hamburger et al., 2005). A mitochondrial ferritin subunit was lately identified in both mammals and insects (Levi et al., 2001; Missirlis et al., 2006). On the contrary to other ferritins, mitochondrial ferritin assembles as homopolymers (Levi et al., 2001). When cells exhibit an iron deficiency, iron can be released from the ferritin. However, an excess of free iron may cause substantial damage to lipids, DNA, and proteins through the generation of highly reactive hydroxyl radicals (Zecca et al., 2004b; Angelova and Brown, 2015). Therefore, strict regulation of iron storage is essential to maintain cellular homeostasis and integrity. *Drosophila* has been successfully used as a model to evaluate the impact of iron storage deregulation on cell physiology and animal behavior. Notably, iron metabolism has been linked to circadian rhythms (Freeman et al., 2013; Rudisill et al., 2019), the autosomal recessive disease Friedreich’s ataxia (Navarro et al., 2015; Soriano et al., 2016), neurodegenerative diseases and age-associated defects (Xun et al., 2008; Rival et al., 2009; Kosmidis et al., 2011, 2014; Tang and Zhou, 2013).

Cells use two main cytosolic degradative processes: the ubiquitin-proteasome pathway (UPS) and the autophagy-lysosomal pathway. While the UPS is specialized in the degradation of monomeric, short-lived proteins; autophagy has the potency to degrade large protein complexes and organelles (Korolchuk et al., 2010; Nam et al., 2017). Autophagy is divided into three different processes that differ by the way substrates are being delivered to the lysosome for degradation. Chaperone-mediated autophagy and microautophagy are defined by their ability to transfer proteins directly to the lysosomes through pores or membrane invagination respectively (Tekirdag and Cuervo, 2017). However, macroautophagy (referred to as autophagy) requires the isolation of cytoplasmic content into double-membraned autophagosomes that eventually fuse with the lysosomes (Yin et al., 2016). The molecular components involved in autophagy progression are highly conserved among Eukaryotes and most of these proteins have orthologs in *Drosophila* (Mulakkal et al., 2014; Bhattacharjee et al., 2019). The complexes of Atg (Autophagy) proteins regulating the formation of autophagosomes are well-conserved and characterized. One essential component of this machinery is the protein Atg8a (LC3 in mammals), which is cleaved and lipidated before anchoring into the autophagosomal membrane (Nagy et al., 2015). Atg8a is essential to the recruitment of other components of the autophagic machinery, as well as for the selection of receptors and their cargoes for selective degradation (Alemu et al., 2012; Wild et al., 2014; Schaaf et al., 2016). The best known selective cargo receptor in *Drosophila* is Ref(2)P (homologous to mammalian p62/SQSTM1) (Nezis et al., 2008; De Castro et al., 2013; Bartlett et al., 2014; Nagy et al., 2014). Selective autophagy can also contribute to the regulation of ferritin turnover (Hou et al., 2016; Gatica et al., 2018). The selective degradation of ferritin by autophagy is referred to as ferritinophagy and requires the cargo receptor NCOA4 in mammals (Mancias et al., 2014, 2015); no homologous receptor has been yet identified in *Drosophila*.

It has been extensively shown that autophagy declines during aging. Indeed, essential autophagy genes are transcriptionally down-regulated during healthy aging (Lipinski et al., 2010; Schultz et al., 2013; Omata et al., 2014). Accumulation of damaged proteins and organelles also constitutes a hallmark of numerous age-associated neurodegenerative disorders (Nixon, 2017; Colacurcio et al., 2018). Alteration of autophagy has been identified as an early onset feature in Alzheimer’s disease-affected neurons (Zare-Shahabadi et al., 2015). However, the interplay between autophagy, iron and neurodegeneration is poorly understood.

In the present study, we used the model organism *Drosophila melanogaster* to investigate the effect of aging and autophagy disruption on the load of iron in the brain. We show that iron and holoferritin (where ferritin – Fer1HCH and Fer2LCH heteropolymer – protein surrounds an iron oxide core) accumulate in the brain from old flies regardless of their autophagy status, suggesting that autophagy is not essential to regulate total iron levels in the *Drosophila* brain. The spectrum of iron phases present is unchanged within the limits of detection for wild-type as a function of aging, but there is evidence of a distinct iron fraction in the autophagy-deficient fly brain, consistent with a proportional elevation in an iron–sulfur phase. This may, in turn, indicate disrupted mitochondrial iron homeostasis (Rouault and Tong, 2005).

## Materials and Methods

### *Drosophila* Stocks and Maintenance

Flies were maintained on standard yeast-cornmeal medium at 25°C, 70% humidity with a 12 h light-dark cycle. The following fly strains were used: wild-type *w*^1118^ (BDRC #3605), Atg8a-deficient *Atg8a*^*KG*07569^ (gift from Dr. Gabor Juhasz), Atg7-deficient *Atg7*^Δ77^ and *Atg7*^Δ14^/*CyO* (Juhasz et al., 2007) (gift from Dr. Gabor Juhasz), *Fer1HCH*^*G*188^/*TM3* (DGRC #110-620; this line encodes a GFP-tagged version of the Fer1HCH subunit) (Missirlis et al., 2007), *hml*(delta)*-GAL4 UAS-eGFP* (BDRC # 30140) and *hml*(delta)*-GAL4 UAS-eGFP UAS-hid/CyO* (gift from Dr. François Leulier). For the generation of Atg7-deficient flies, virgin females *Atg7*^Δ14^/*CyO* were crossed with males *Atg7*^Δ77^ and the progeny lacking balancer chromosome was collected after hatching. Stocks were backcrossed to *w*^1118^ to isogenise the genetic background.

### Aging and Lifespan Measurement

For all experiments, age-matched adult male flies were used. Flies were collected within 24 h of hatching and aged in cohorts of 20 individuals. Flies were transferred every 2–3 days on fresh medium until collection after 1 week, 1 or 2 months. Because of their shorter lifespan, old autophagy-deficient flies were collected at 1 month.

### Generation of Hemizygous GFP-Fer1HCH Expressing Flies

Homozygous virgin females wild-type (*w*^1118^) or autophagy-deficient (*Atg8a*^*KG*07569^) were crossed with males *Fer1HCH*^*G*188^/*TM3*. From the progeny, only hemizygous males *w*^1118^/*Y;* Fer1HCH^*G*188^/+ and *Atg8a*^*KG*07569^/*Y;* Fer1HCH^*G*188^/+ were collected, and aged or fed as mentioned where appropriate in the figure legends.

### Feeding With Iron or Bortezomib-Supplemented Diets

Adult males were selected within 24 h from hatching and placed onto Nutri-Fly Instant *Drosophila* Medium (Genesee Scientific, 66–117) prepared in water supplemented with 1 mM FAC (ferric ammonium citrate) or 20 mM bortezomib in DMSO (#2204 Cell Signaling Technology). Flies were flipped onto freshly made food every day for 5 days. The same diet without FAC or with 0.002% DMSO were used as respective control for regular diets.

### Protein Extraction From Adult *Drosophila* Heads and Bodies

Age-matched adult males were flash frozen in liquid nitrogen. Flies were decapitated by short burst vortexing in 15 mL tubes. Heads, bodies, and appendices were separated using sieves (no. 25 and no. 40) chilled with liquid nitrogen beforehand. Heads were collected in microcentrifuge tubes and homogenized in ice-cold lysis buffer (20 mM Tris pH 7.5, 137 mM NaCl, 1% Triton X-100, 1% glycerol) supplemented with complete protease inhibitor cocktail (cOmplete^TM^, Mini, EDTA-free Protease Inhibitor Cocktail; Sigma-Aldrich, 04693159001 Roche) and 50 mM *N*-ethylmaleimide (Sigma-Aldrich, E3876). Protein concentrations were determined using Bradford assay.

### Hemocyte Ablation

The cell-specific ablation of mature hemocytes was performed by crossing virgin females *Fer1HCH^*G188*^/TM3* with males *hml*(delta)*-GAL4 UAS-eGFP UAS-hid/CyO*. As a control, virgin females *Fer1HCH^*G188*^/TM3* were crossed with males *hml*(delta)*-GAL4 UAS-eGFP/CyO*. From the progeny, adult flies lacking the balancer chromosomes were selected and aged for 5 days onto regular diet before collection of their hemolymph.

### Hemolymph Collection

Immediately before hemolymph collection, anesthetized flies were surface sterilized by dipping them briefly in 70% ethanol. Excess ethanol was blotted off on filter paper. Flies were punctured with a tungsten needle in their thorax and immediately placed in a collection tube on ice. Collection tubes were made by piercing through the bottom of a 0.5 mL centrifuge tube with a 25G needle and placing it into a 1.5 mL centrifuge tube. A total of 40 punctured flies per genotype were pooled per collection tube. Hemolymph was isolated by centrifugation at 5000 rpm for 5 min at 4°C. Collected hemolymph samples were then diluted in Laemmli loading buffer and heated for 5 min at 95°C.

### *In gel* Iron Staining

Protein extracts were prepared in 2x concentrated non-denaturing/non-reducing loading buffer (62.5 mM Tris-HCl pH 6.8, 25% glycerol, 1% bromophenol blue). Protein concentrations were determined using Bradford assay; 20 μg of total protein for each sample was separated on 6% native-PAGE gel in ice-cold running buffer (25 mM Tris, 192 mM glycine) after pre-run of the gel for 30 min at 100 V. Following protein separation, the gel was stained for 48 h with Prussian blue staining solution (10% K_4_Fe(CN)_6_, 350 mM HCl) at room temperature with gentle agitation. After washes in ultrapure water, holoferritin was visible as blue bands. All the glassware and tanks were acid-rinsed (1% HCl in ultrapure water) and left to air dry before use.

### Western Blotting and Antibodies

Protein extracts were prepared in Laemmli loading buffer containing 2.5% beta-mercaptoethanol (except for experiment in non-reducing condition where no beta-mercaptoethanol was added) and heated for 5 min at 95°C before separation of 20 μg total proteins on 8 or 12% SDS-PAGE gels. Separated proteins were transferred onto nitrocellulose or PVDF membranes. The membranes were blocked in TBS (Tris-buffered saline; 50 mM Tris-Cl, pH 7.6, 150 mM NaCl), 0.1% Tween-20, 5% non-fat milk. The following antibodies were used: anti-GFP (Santa Cruz sc-9996, 1:1,000), anti-GABARAP/Atg8a (Cell Signaling Technology No. 13733, 1:2,000), anti-Ref(2)P (Abcam ab178440, 1:1,000), anti-β actin (Abcam ab8227, 1:2,000), anti-α tubulin (Sigma-Aldrich T5168, 1:40,000), HRP-coupled secondary antibodies anti-rabbit and anti-mouse (Thermo Scientific No. 31460 and 31450, 1:10,000). Signals were developed using the ECL detection reagents (Amersham, RPN2209).

### Immunocytochemistry

Dissected brains from adult males *Fer1HCH*^*G188*^ were fixed for 30 min in 4% paraformaldehyde in 1x PBS (phosphate buffered saline; 137 mM NaCl, 10 mM Phosphate, 2.7 mM KCl, pH 7.4). The brains were permeabilized for 1 h in permeabilization buffer (0.1% Triton X-100, 0.3% BSA in PBS) before incubation overnight at 4°C with anti-Brp (DSHB, nc82 supernatant; 1:10 in permeabilization buffer) (Wagh et al., 2006) or anti-Elav (DSHB, Elav-9F8A9 supernatant; 1:100 in permeabilization buffer) (O’neill et al., 1994). Subsequent incubation with an Alexa568-coupled secondary antibody (Sigma No. SAB4600082, 1:500) was conducted in permeabilization buffer for 2 h at room temperature. Nuclei were stained with Hoechst 33342 (1 μg/mL in PBS). All washes were performed with 0.1% Triton X-100 in PBS. Images were captured with a Zeiss LSM880 confocal microscope.

### X-Ray Fluorescence Imaging and Spectroscopy on Isolated Brains

Whole dissected brain from flies at the desired age were dissected using tungsten-coated titanium tweezers in ultrapure deionized water. Dissected brains were mounted onto ultralene film and allow to air dry for a minimum of 2 h (nine brains per slide: three brains per genotype/age).

Specimens were analyzed at the I18 Microfocus Spectroscopy beamline at the Diamond Light Source in Oxford, United Kingdom, using a pair of opposing Si detectors to maximize recovery of the fluorescence emitted from the ultralene-mounted samples. The focused beam was tuned to 10.5 keV, with a beam spot diameter of 60 μm for initial surveys with microfocus X-ray Fluorescence (μXRF), and 20 μm for mapping over the area of each intact brain. The method used here is not as precise in determining absolute concentration as mass spectrometry imaging, but it is non-destructive and highly sensitive to relative differences in concentration between samples (Collingwood and Adams, 2017). Three to nine intact brains were imaged at room temperature for each group of flies. Detector position and acquisition times were kept consistent throughout the experiment to facilitate the subsequent comparative analysis. Subsequently, site-specific X-ray Absorption Near Edge Spectroscopy with a microfocused beam (μXANES) analysis was performed at the iron K-edge, using the 20 μm diameter beam to acquire a spectrum from the iron-richest region in the central brain from each fly, where the region of interest (ROI) was confirmed using the μXRF intensity image for iron. The absorption edge position in energy was calibrated with reference to an iron foil spectrum obtained during the same experiment, with alignment and removal of background being performed using the established workflow in the IFEFFIT Athena software package for XAFS analysis (Ravel and Newville, 2005).

### Genomic DNA Extraction and PCR

Genomic DNA (gDNA) from 15 flies per genotype was extracted using DNeasy Blood and Tissues Kit (Qiagen 69504). PCR amplifications were conducted on 100 ng of gDNA with DreamTaq Green PCR Master Mix (Thermo Scientific K1081) in a Bio-Rad T100 thermal cycler. Samples were loaded on 1% (w/v) agarose gel in 1x TAE (40 mM Tris-base, 20 mM acetic acid, 1 mM EDTA pH 8.0). GelRed (VWR 41003) was used to stain nuclei acids. Primer sequences are listed in Table 1.

**TABLE 1:** Primer sequences used in this study.

**PCR primers**		
HmlΔ forward	CCAACAATTTCCGATTAGCCTAAC	
GAL4 reverse	CGATACAGTCAACTGTCTTTGACC	
pUAST3′	AACCAAGTAAATCAACTGC	
Hid reverse	GAATGGTGTGGCATCATGTGC	
EGFP reverse	CTTGTAGTTGCCGTCGTCCTTGAA	

**RT-qPCR**	**Forward primer sequences**	**Reverse primer sequences**

*fer1hch*	TCTGATCAATGTGCCGACTG	TGGTAGTGGTTGTAGGGCTTG
*fer2lch*	GCCAGAACACTGTAATCACCG	GGCTCAATATGGTCAATGCCA
*Atg8a*	GGTCAGTTCTACTTCCTCATTCG	GATGTTCCTGGTACAGGGAGC
*Atg7*	TCGTGGGCTGGGAGCTAAATA	GGTTTACAGAGTTCTCAGCGAG
*rp49*	AAGAAGTTCCTGGTGCACAACGTG	AATCTCCTTGCGCTTCTTGGAGGA

### RNA Extraction and Real Time (RT)-qPCR

Total RNA extraction was performed on adult males using the PureLinkTM RNA Mini kit (Life Technologies Ambion) according to the manufacturer protocol. For all subsequent steps, 1 μg of RNA was used for each condition. Genomic DNA were digested out using DNase I (Thermo Scientific K1622). Synthesis of cDNA was done using the RevertAid Kit (Thermo Scientific K1622). Relative quantitation of gene expression was performed in an Agilent MxPro4005P qPCR system using the GoTaq qPCR Master Mix (Promega A6002). Primer sequences are listed in Table 1.

## Results

### Ferritin Is Expressed in Adult *Drosophila* Brain

**FIGURE 1:**
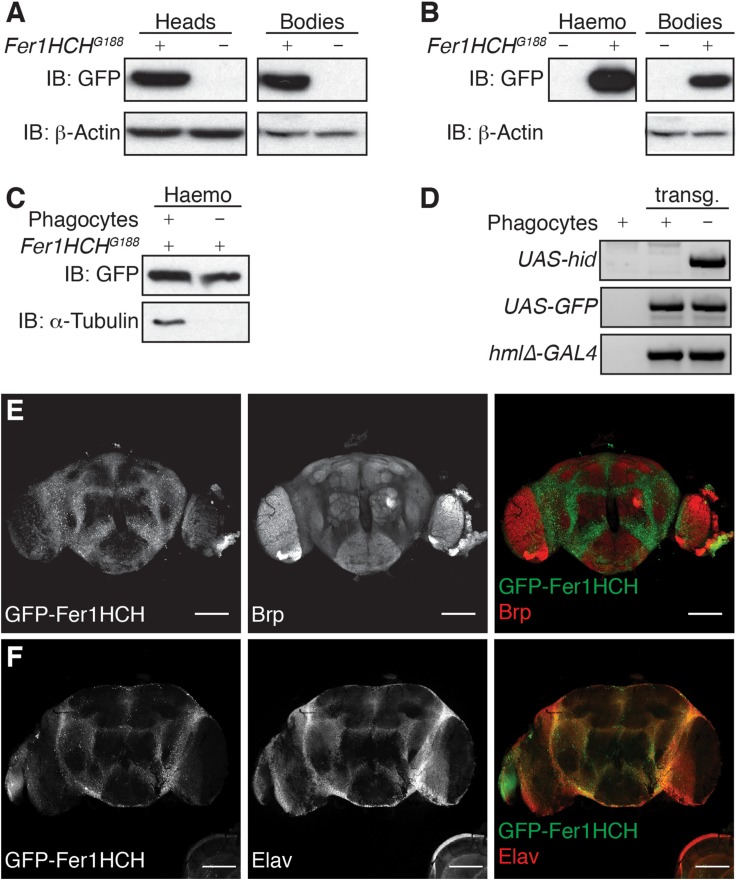
Ferritin heavy chain is expressed in adult *Drosophila* brain. **(A–C)** Western blot analysis of GFP-Fer1HCH in reduced/denaturated protein samples extracted from **(A)** fly heads or bodies; **(B)** hemolymph (Haemo) or bodies drained from their hemolymph (Bodies); **(C)** hemolymph from flies depleted (–) or not (+) of their phagocytes. **(D)** Gel electrophoresis on PCR products from gDNA extracted from transgenic (transg.) flies used in **(C)**, gDNA from *w*^1118^ wild-type flies was used as negative control. **(E,F)** Confocal imaging of an adult *Drosophila* brain expressing GFP-Fer1HCH (left panel, green in merged panel) and stained for either **(E)** Bruchpilot (Brp) or **(F)** Elav (middle panel, red in merged channel). For all the experiments, 1-week-old adult males were used.

It has been previously shown that ferritin is expressed in various tissues in *Drosophila*. The protein expression of a GFP-knock-in mutant for *fer1hch* has been used to show the localization of Fer1HCH protein in different tissues and organs, including the larval brain where it accumulates primarily in the optic lobes and notochord (Georgieva et al., 2002; Mehta et al., 2009). However, no information is readily available concerning the localization of the GFP-Fer1HCH in adult fly brain. To evaluate the expression of GFP-Fer1HCH in the head of adult flies, we performed western blot on lysates from isolated heads and bodies from a fly strain expressing GFP-tagged Fer1HCH due to the genomic insertion of GFP between the first and second exons of the gene (*Fer1HCH*^*G188*^ flies) (Missirlis et al., 2007). Wild-type flies were used as a negative control. As expected from previous studies, GFP-Fer1HCH was detected in samples from both heads and bodies (Figure 1A).

*Drosophila* ferritin is known to be secreted in the hemolymph, which is analogous to vertebrates’ blood, and remains in direct contact with tissues. In order to evaluate whether the GFP-Fer1HCH protein we detected in *Drosophila* heads corresponds to secreted or cytosolic ferritin, we extracted the hemolymph from adult *Fer1HCH*^*G188*^ flies. As expected, GFP-Fer1HCH strongly accumulates in the hemolymph, but also remains present in the bodies after draining their hemolymph (Figure 1B). It was shown that GFP-Fer1HCH accumulates in hemocytes (Gonzalez-Morales et al., 2015). To make the distinction between ferritin which is secreted in the hemolymph and expressed in the hemocytes, we compare the level of GFP-Fer1HCH protein in the hemolymph from flies lacking mature hemocytes (phagocytes) and flies with a normal hemocytes pool (Figures 1C,D). Tubulin was used as a marker for the successful ablation of the phagocytes (Figure 1C) and the presence of the relevant transgenes was validated by PCR on gDNA (Figure 1D). We observed a moderate reduction of GFP-Fer1HCH in the hemolymph from the flies lacking phagocytes, meaning that most of the ferritin in the hemolymph corresponds to secreted ferritin (Figure 1C).

To confirm that ferritin detected in the adult *Drosophila* head comes mainly from the tissues rather than the surrounding hemolymph, we used fluorescence microscopy to establish the distribution of ferritin in *Drosophila* adult brain. We observed that GFP-Fer1HCH is expressed in the cell bodies surrounding the neuropil, marked using an antibody against the presynaptic protein Bruchpilot (Brp) (Wagh et al., 2006) (Figure 1E). The localization of GFP-Fer1HCH also matches the expression of Elav, a neuron-specific protein (Figure 1F).

Taken together, the above results show that ferritin heavy chain is expressed in the brain of adult *Drosophila* fly and accumulates in the neuronal cell bodies.

### Decline of Autophagy Induces Accumulation of High Molecular Weight Ferritin Heavy Chain

**FIGURE 2:**
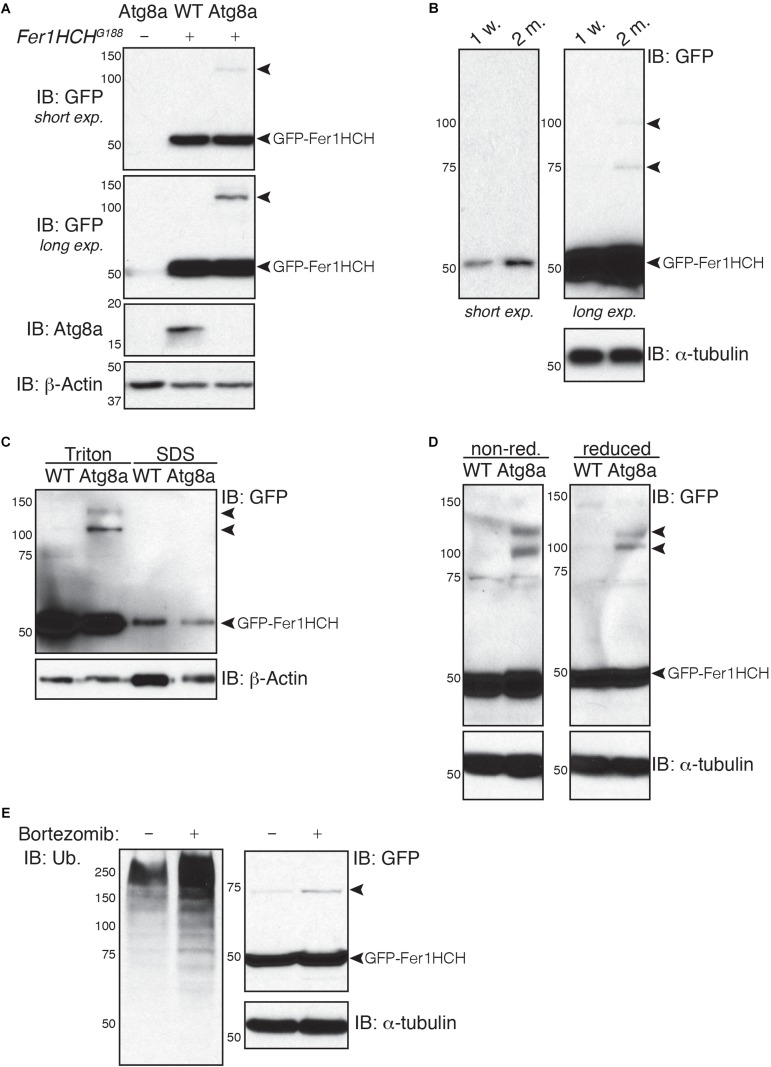
Soluble high molecular weight heavy-chain ferritin accumulates in autophagy-deficient fly heads. **(A,B)** Western blot analysis of GFP-Fer1HCH after samples reduction/denaturation in total protein lysates from **(A)** 1-week old wild-type (WT) and Atg8a mutant flies or **(B)** 1-week or 2-months old wild-type flies. **(C)** Western blot analysis of GFP-Fer1HCH in soluble (Triton) and insoluble/aggregated (SDS) proteins fraction from 1-week old wild-type (WT) and Atg8a mutant fly heads. **(D)** Western blot analysis of GFP-Fer1HCH in total protein lysates from 1-week old wild-type (WT) and Atg8a mutant fly heads prepared in either reduced or non-reduced conditions before SDS-PAGE and western blotting. **(E)** Western blot analysis of GFP-Fer1HCH in total protein lysates from Fer1HCHG188 fly heads after feeding for 6 days on 10 μM bortezomib or vehicles. Membranes were probed for GFP, Atg8a **(A)**, ubiquitinated proteins **(E)**; β-actin **(A,C)** or α-tubulin **(B,D,E)** were used as loading control. Arrowheads show high molecular weight bands of GFP-Fer1HCH. The terms ‘short exp.’ and ‘long exp.’ refer to the duration of film exposure on the membrane before developing. A longer exposure (long exp.) was necessary for the observation of the high molecular weight GFP-Fer1HCH which are less abundant than 50 kDa GFP-Fer1HCH.

Various studies have demonstrated that autophagy, which declines with age, is implicated in the degradation of ferritin and iron turnover (Asano et al., 2011; Mancias et al., 2014; Ott et al., 2016). To evaluate whether autophagy and aging affect the level of ferritin in adult *Drosophila* heads, we made use of the GFP-Fer1HCH expressing flies. First, extracts from adult heads of wild-type or Atg8a mutant males heterozygous for the *Fer1HCH*^*G188*^ allele were analyzed. Atg8a mutant fly heads were used as a negative control for GFP-Fer1HCH expression. Western blots probed with anti-GFP antibody revealed the presence of a band at the expected size of 50 kDa consistent with the fusion of the 27 kDa GFP protein to the 23 kDa Fer1HCH chain (Figure 2A). No noticeable difference was observed between wild-type and autophagy mutant. However, 1-week old Atg8a mutant fly head samples, but not young age-matched wild-type, exhibited the accumulation of a higher molecular weight band around 120 kDa (Figure 2A). Similarly, we noted the presence of higher molecular weight bands in old (2-months old) *Fer1HCH*^*G188*^ male fly heads that were not detected in young (1-week old) wild-type flies (Figure 2B).

To test whether this high molecular weight ferritin in Atg8a mutant and aged wild-type fly heads corresponds to aggregates, we performed a differential detergent protein extraction (Nezis et al., 2008; Simonsen et al., 2008; Jacomin and Nezis, 2019). Soluble proteins from fly heads were first extracted in a 0.1% Triton X-100 lysis buffer. The pellets, containing aggregated proteins, were then broken down by sonication in a 2% SDS lysis buffer. Samples were reduced and denaturated before separation by SDS-PAGE. We observed that 50 kDa GFP-Fer1HCH is predominantly located in the soluble fraction (Triton). High molecular weight ferritin heavy chains were solely detected in the soluble fraction and were excluded entirely from the insoluble fraction (SDS) (Figure 2C). Some proteins can form oligomers that can be identified using reduced and non-reduced lysis condition. We compared the effect of reducing agent on the formation of higher molecular weight GFP-Fer1HCH. Lysis of wild-type and Atg8a mutant fly heads was performed in lysis buffer supplemented with 50 mM *N*-ethylmaleimide to prevent the formation of new disulfide bond during the lysis procedures. Loading samples were then prepared by boiling in SDS-loading buffer in the presence (reduced) or absence (non-reduced) of 2.5% β-mercaptoethanol (Figure 2D). The preparation of the samples from in non-reduced condition had no effect on the accumulation of the high molecular weight GFP-Fer1HCH which was consistently observed in 1-week old autophagy mutant (but not age-matched wild-type) fly heads samples. To evaluate the implication of the proteasome in the clearance of ferritin, we have fed flies expressing GFP-Fer1HCH with bortezomib – an inhibitor of the proteasome – and looked at the accumulation of GFP-Fer1HCH high molecular weight band; no significant effect was observed (Figure 2E). The efficiency of bortezomib to block the proteasome was checked by probing the membrane with an antibody against ubiquitinated proteins, which accumulate when the proteasome is blocked (Figure 2E).

Taken together, these results show that ferritin does not form aggregates in *Drosophila* head when autophagy is impaired.

### Holoferritin Is Not Affected in Autophagy Mutant Fly Heads

**FIGURE 3:**
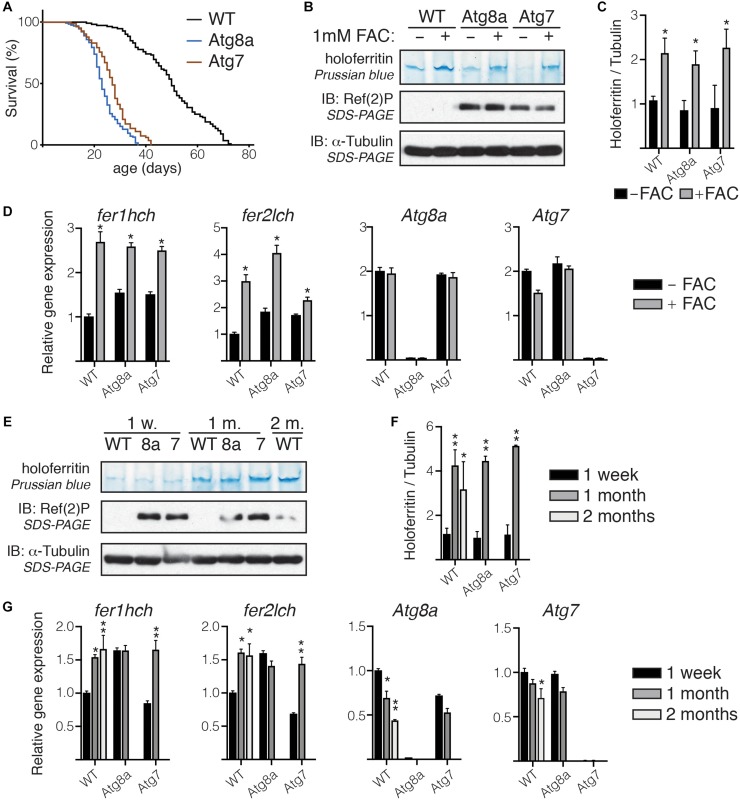
Holoferritin accumulation in fly head. **(A)** Lifespan of a 100 wild-type and Atg8a and Atg7 mutant flies reared in standard conditions. **(B)** *In gel* staining for holoferritin in protein lysates from wild-type and Atg8a and Atg7 mutant flies fed on either normal diet or diet supplemented with 1 mM FAC. **(C)** Relative quantity of holoferritin normalized to loading control. **(D)** Analysis by RT-qPCR of mRNA level for *fer1hch*, *fer2lch*, *Atg8a*, and *Atg7* in flies fed on either normal or FAC-supplemented diet. **(E)** *In gel* staining for holoferritin on protein lysates from wild-type (WT) Atg8a (8a) and Atg7 (7) mutant fly heads at 1-week, 1-month and 2-month old. **(F)** Relative quantity of holoferritin normalized to loading control. **(G)** Analysis by RT-qPCR of mRNA level for *fer1hch*, *fer2lch*, *Atg8a*, and *Atg7* in aged flies. The bar charts show mean ± SD. Statistical significance was determined using one-way ANOVA, ^*^P < 0.05, ^∗∗^*P* < 0.01. Western blots on denaturated samples were probed for Ref(2)P as an autophagy-deficiency control and α-tubulin as a loading control.

It has been well-documented that iron bioavailability and ferritin levels are correlated. We have verified that we can detect changes in holoferritin in wild-type and autophagy-deficient *Drosophila* by feeding adult flies on either normal diet or on a diet supplemented in iron in the form of FAC. Flies lacking either Atg8a or Atg7, two major regulators of autophagy, were used as autophagy-deficient *Drosophila*. We performed Prussian blue staining for holoferritin in protein samples from whole adult flies separated on native-PAGE. As expected, holoferritin accumulated in flies fed on iron-supplemented diet (Figures 3B,C) and correlated with an increase in the mRNA expression level of *fer1hch* and *fer2lch*, while Atg8a and Atg7 remained unchanged (Figure 3D).

We then performed *in gel* Prussian blue staining on protein lysates prepared from wild-type or autophagy-deficient fly heads collected from age-matched flies at 1-week, 1-month, or 2-months old. Because of their significantly shorter lifespan, samples from old Atg8a and Atg7 mutant flies were collected at 1-month old only, solely wild-type samples were collected at 2-month old (Figure 3A). We observed that holoferritin accumulated in protein samples from heads isolated from old flies, regardless of their autophagy-deficiency status. No difference was observed in heads from young autophagy-deficient flies when compared to age-matched wild-type (Figures 3E,F). The quantity of holoferritin was normalized against α-tubulin, used as a loading control, from the same samples separated by SDS-PAGE after denaturation and reduction of the samples in Laemmli loading buffer and boiling at 95°C. Gene expression of *fer1hch* and *fer2lch*, as well as *Atg8a* and *Atg7*, was accessed by RT-qPCR. Wild-type and Atg7 mutant flies showed an increased level in *fer1hch* and *fer2lch* as a result of their respective aging, which correlated with the accumulation of holoferritin, while no significant change was observed in Atg8a mutants (Figure 3G). As expected, Atg8a expressing is reduced in old wild-type flies (Simonsen et al., 2008; Omata et al., 2014).

Altogether, these results suggest that the accumulation of holoferritin during the course of aging in *Drosophila* head is independent of autophagy.

### Ferric Iron Accumulates in the Brain of Aged Flies

**FIGURE 4:**
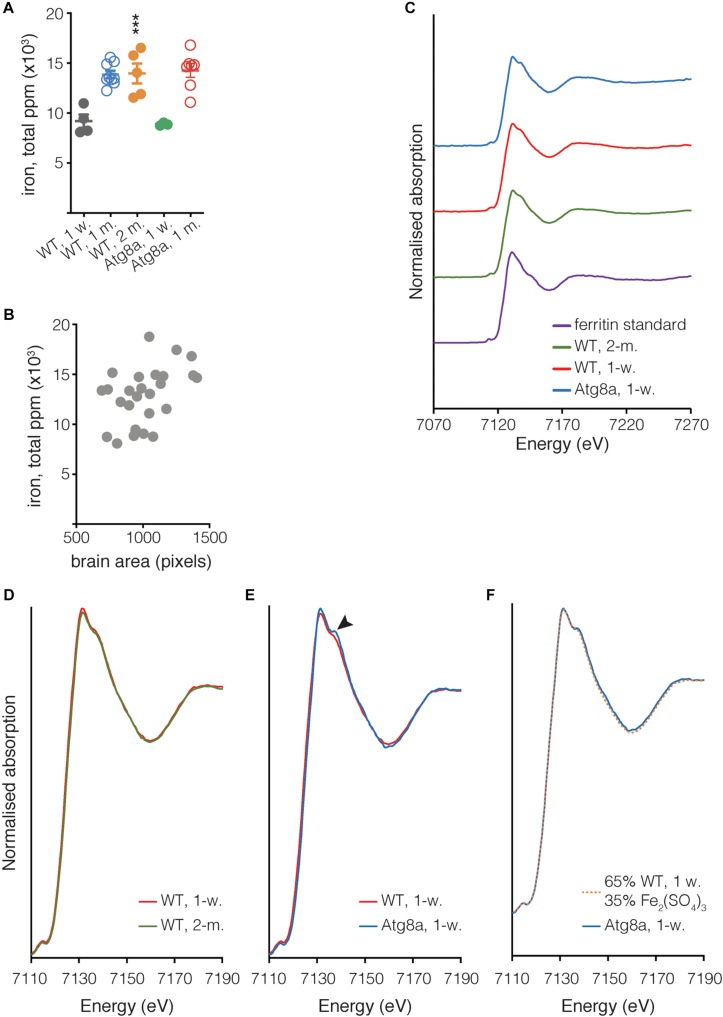
Quantification of iron in isolated *Drosophila* brain. **(A)** The total quantity of iron per whole fly brain was measured from complete brain images obtained using μXRF at 20 μm resolution. Individual biological samples are shown as circles. Open circle data were acquired during a different experiment than close circle data. Bar represents mean ± SD. Statistical significance was determined using one-way ANOVA; significant values are indicated above the bars, ^∗∗∗^*P* < 0.001. **(B)** Correlation between the area of the brain in pixels (px) and the quantity of iron. Spearman’s rank correlation coefficient ρ = 0.3311. **(C)** Fe K-edge XANES from *Drosophila* and ferritin standard, with spectra vertically offset for clarity. **(D,E)** Overlay of spectra shown in **(C)**, focussing on the region where the spectra from Atg8a and wild-type differ (arrowhead). **(F)** Linear combination fitting of Atg8a spectrum. The Atg8 spectrum was shown to be consistent with that of the wild-type model + Fe(III) sulfate (Fe_2_(SO_4_)_3_), with a χ^2^ value of 0.01 obtained for the fit. All XANES spectra were subjected to an edge-step normalization and flattened using Athena fitting software prior to fitting.

X-ray fluorescence microscopy was previously demonstrated to be a robust way to image and quantify biometals in the non-mammalian model organisms, *Drosophila* and *Caenorhabditis elegans* (Lye et al., 2011; Jones et al., 2015; Ganio et al., 2016). Therefore, we used synchrotron μXRF imaging to measure the concentration of iron in the brain of *Drosophila*. Elemental maps were collected from whole brains dissected from young and old wild-type flies and young autophagy-deficient flies (*Atg8a* mutant). Three to nine entire brains per fly group were imaged at 20 μm resolution, providing an excellent signal to noise for the elements of interest. The data collected were used to quantify and compare iron concentrations (ppm) between each group. The quantification was done by first defining the ROI encompassing each brain based on the iron distribution map, and calculating the mass fraction for elements of interest after subtraction of the background signal (accounting for any background scatter reaching the detector, including any signal from the ultralene). As the present study focuses on iron, the other elements simultaneously acquired in the XRF imaging merit further investigation and will be the subject of future work. We observed that iron accumulated as a function of aging in the wild-type and autophagy-deficient brains (Figure 4A). Checking the relationship between the area mapped and the total metal ion signal confirmed that there was no correlation between the size of the brain and the amount of iron as shown by the Spearman’s rank correlation coefficient ρ = 0.3311 (Figure 4B).

In addition to determining the elemental distribution of iron by μXRF, we performed site-specific microfocus X-ray Absorption Near Edge Structure (μXANES) spectroscopy analysis to obtain information about the dominant iron phase(s) present in the central brain region where iron deposition was the highest for each of the three groups. The μXANES spectra from wild-type and autophagy deficient flies incorporate the sum of contributions from the iron phases present at the iron-richest region in the central brain, including signal from the mineralized iron in holoferritin, typically a ferrihydrite-like hydrated iron oxide (Collingwood and Telling, 2016); the spectrum from the iron in purified horse spleen ferritin is included for comparison (Figure 4C). The μXANES spectra from these iron-rich sites in the 1-week and 2-months old wild-type flies are indistinguishable in this experiment (Figure 4D), whereas comparison of 1-week old wild-type and Atg8a-deficient reveals an additional feature in the autophagy-deficient fly at 7138 eV (Figure 4E). Linear combination fitting was undertaken for the 1-week old Atg8a-deficient fly spectrum using the 1-week wild-type spectrum and a range of reference standards (including those measured recently at the same beamline: iron metal reference foil, ferric sulfate, ferric and ferrous chloride, ferric citrate, horse spleen ferritin, previously-acquired iron nitride, and iron oxide standards including ferrihydrite, magnetite, and wustite). The best fitting result indicated that the feature can be well-accounted for by including a ferric sulfate reference standard (Figure 4F), suggesting that approximately 1/3 of the signal might be attributed to iron–sulfur complexes, and 2/3 attributed to the spectrum of iron phases found in the central brain of the wild-type fly.

## Discussion

During the course of aging, the load of iron in the brain increases significantly, possibly due to decreased efficiency of the iron homeostasis system. Neurodegenerative pathologies associated with aging, such as Parkinson’s or Alzheimer’s diseases, have previously been associated with changes in iron homeostasis (Zecca et al., 2004a; Ward et al., 2014).

Our data corroborate previous studies of iron accumulation in the brain during aging in *Drosophila* (Massie et al., 1985, 1993). However, it was surprising to observe that neither iron nor ferritin heavy-chain levels are affected in autophagy-deficient flies. Indeed, an increase in iron in those flies was anticipated because of the recent studies pinpointing at the impact of autophagy on iron mobilization and recycling (Kurz et al., 2011; Mancias et al., 2014; Ott et al., 2016). The lack of accumulation of iron and holoferritin in autophagy-deficient flies at any age, matched to the age of wild-type individuals, suggests that autophagy is either not required or plays a minor role in the turnover of ferritin and iron during the course of aging in *Drosophila* brain. A recent study showed that lysosomal trafficking of ferritin could be independent of macroautophagy (Goodwin et al., 2017). Nonetheless, we noticed the presence of supernumerary GFP-Fer1HCH bands in the heads of young Atg8a-deficient flies while the main GFP-Fer1HCH band remained unchanged in age-match wild-type fly heads. This high molecular weight GFP-Fer1HCH probably corresponds to a non-functional form of ferritin heavy-chain as there is no accumulation of holoferritin in these flies as demonstrated by Prussian Blue *in gel* staining and μXRF. Autophagy has been extensively described for its role in the degradation of protein aggregates, and ferritin has been shown to be degraded by autophagy (Hyttinen et al., 2014; Mancias et al., 2014). However, using a differential-detergent protein fractionation protocol, we have observed that high molecular weight GFP-Fer1HCH bands do not correspond to insoluble aggregates. A shift in protein molecular weight could be associated with post-translational modifications. A study has shown that both ferritin subunits are ubiquitinated in muscles from a rat model of Amyotrophic Lateral Sclerosis (Halon et al., 2010). Ferritin has also been detected as being pupylated (prokaryotic homolog of ubiquitination) in the bacterium *Corynebacterium glutamicum* (Kuberl et al., 2016). Ferritin is also known to be glycosylated in mammals and insects; notably, secreted ferritin L has been shown to be N-glycosylated in culture hepatocytes (Cragg et al., 1981; Ketola-Pirie, 1990; Ghosh et al., 2004; Cohen et al., 2010). Therefore, it is possible that high molecular weight Fer1HCH corresponds to a modified, soluble form of the protein.

It was previously shown that iron storage increases significantly with age in both mammals and insects (Massie et al., 1985; Zecca et al., 2001). The control of ferritin subunit synthesis frequently occurs at the translational level. Ferritin mRNAs contain an iron-responsive element (IRE) in 5′ UTR that can be recognized by iron regulatory proteins (IRPs). Depending on iron availability, the translation of ferritin subunits is modulated by the binding or releasing of the IRPs to the mRNA (Gray and Hentze, 1994; Lind et al., 1998; Missirlis et al., 2007). Interestingly, no noticeable increase in the quantity of GFP-Fer1HCH with age was observed in the fly brain while the iron levels and holoferritin levels were significantly increased. Most of the studies aiming at elucidating the IRE/IRP-dependent regulation of ferritin are based on supplementation of animal food with iron or chelators. It is possible that such changes in the diet have more drastic effects on the iron uptake by the cells than that which would occur under physiological conditions. It is also possible that intestinal ferritin is more prone to transcriptional regulation as the gut is the first organ to be affected by dietary iron.

The evidence for an additional minor peak at 7138 eV in the iron absorption spectrum for autophagy deficient flies, but not in wild-type, is consistent with signal contribution from an iron–sulfur-rich material. The good fit achieved with the inclusion of ferric sulfate does not exclude other possibilities; we note that iron-phosphorus-containing material can also exhibit a peak in this energy region. However, examination of the XRF signal at the sites where the XANES spectra were acquired indicate that sulfur was significantly more abundant than phosphorus, and that while iron and sulfur levels at sites of XANES acquisition were equivalent for wild-type and autophagy mutant, the phosphorus level at the XANES site in the autophagy deficient fly was lower (approximately 1/5^*th*^) of that measured in wild-type. Therefore, it is more likely that the additional feature in the autophagy mutant is associated with iron–sulfur than with iron–phosphorus. Iron–sulfur clusters might account for this signal, a ubiquitous class of metalloproteins involved in many regulatory processes, including mitochondrial iron homeostasis (Rouault and Tong, 2005).

In summary, we have shown that holoferritin accumulates in the brain from old flies but not young Atg8a-deficient flies, suggesting that macroautophagy is not a dominant process in ferritin and iron turnover in the *Drosophila* adult head. The origin of increased iron in the brain during the course of aging in *Drosophila* brain remains unclear but appears not to be related to, or sufficient to induce the synthesis of, ferritin heavy-chain. In addition, our work provides evidence of the feasibility to accurately detect variation in biometal levels and distributions in intact isolated adult *Drosophila* brain, thus opening new fields of investigation for normal and pathological aging. Most neurodegenerative diseases are linked to altered metabolism of biometals, including iron, in the brain. *Drosophila* has been successfully used as model for a broad range of neuropathologies. Further studies combining those readily-available model strains with synchrotron spectromicroscopy methods should contribute to uncovering the relationships between disrupted metabolism of biometals and neurodegeneration.

## Author Contributions

A-CJ, KG, JB, VT-T, and JC performed the experiments and analyzed the data. KG, JB, VT-T, and JC provided material, expertise, and technical help in synchrotron X-ray fluorescence microscopy data acquisition and analysis. A-CJ, IN, and JC designed the study. A-CJ wrote the manuscript and analyzed the data. All authors read and contributed to the manuscript.

## Conflict of Interest Statement

The authors declare that the research was conducted in the absence of any commercial or financial relationships that could be construed as a potential conflict of interest.
